# A Meta-Analysis Comparing Sublay and Onlay Mesh Repair in Incisional Hernia Surgery Based on Surgical Outcomes

**DOI:** 10.7759/cureus.87541

**Published:** 2025-07-08

**Authors:** Wishal Shaukat, Malik Asfand Yar, Zulfiqar Ali, Tahir Aslam, Saud Hussain, Qamar Yasmeen, Kamran Hyder Abbasi, Mohammad Shiraz

**Affiliations:** 1 General Surgery, University Hospitals Birmingham, Birmingham, GBR; 2 Acute Medicine, Queen Elizabeth Hospital, University Hospitals Birmingham (UHB) National Health Services (NHS) Foundation Trust, Birmingham, GBR; 3 General Surgery, Rashid Latif Medical College, Lahore, PAK; 4 General Surgery, Institute Bolan Medical College, Quetta, PAK; 5 General Surgery, Shalamar Institute of Health Sciences, Lahore, PAK; 6 Biochemistry, Niazi Medical and Dental College, Sargodha, PAK; 7 General Surgery, Liaquat University of Medical and Health Sciences, Jamshoro, PAK; 8 General Surgery, Peterborough City Hospital, North West Anglia Foundation Trust, Peterborough, GBR

**Keywords:** incisional hernia, onlay mesh repair, postoperative recovery, sublay mesh repair, surgical outcomes

## Abstract

Incisional hernia is a common surgical complication that often requires mesh-based repair. Among the various techniques, sublay (retrorectus) and onlay mesh placements are frequently employed, but their relative effectiveness in terms of surgical outcomes remains debated. This meta-analysis aimed to compare sublay and onlay mesh repair techniques in incisional hernia surgery based on key postoperative outcomes. A systematic search was conducted across PubMed, Cochrane Central Register of Controlled Trials (CENTRAL), Scopus, ProQuest, and Google Scholar for studies published in English between January 2010 and March 2025. Eligible studies included randomized controlled trials and prospective comparative studies directly comparing sublay and onlay mesh repair techniques and reporting on at least one predefined surgical outcome. Six studies involving a total of 468 patients were included in the analysis. Primary outcomes assessed included hernia recurrence, postoperative complications, surgical site infection (SSI), and seroma formation. Secondary outcomes included operative time and length of hospital stay. Data were analyzed using a random-effects model with odds ratios (OR) and mean differences (MD) reported along with 95% confidence intervals (CI). The meta-analysis revealed that sublay mesh repair was consistently associated with lower hernia recurrence rates, though the differences did not reach a statistical significance. Sublay repair also demonstrated significantly fewer postoperative complications in multiple studies, particularly in terms of reduced seroma formation and SSI. Furthermore, hospital stay was significantly shorter in the sublay group in two of the three studies reporting this outcome. Onlay repair, however, was associated with a shorter operative time. In conclusion, sublay mesh repair offers superior outcomes in terms of recurrence, complications, seroma, and hospital stay, suggesting it as the preferred approach for incisional hernia repair in appropriate clinical settings.

## Introduction and background

Incisional hernias represent a significant subset of anterior abdominal wall hernias and pose a substantial clinical challenge due to their high prevalence and potential for postoperative complications [1]. Among the various techniques developed for their repair, mesh-based reinforcement has emerged as the standard of care, offering superior outcomes compared to primary suture repair [2]. Polypropylene mesh, introduced in 1962, has become the cornerstone of hernia surgery, with different techniques of mesh placement evolving over time, including onlay, inlay, sublay, preperitoneal, and intraperitoneal approaches [3]. Despite advancements, the optimal mesh placement technique remains a topic of ongoing debate, particularly between the onlay and sublay methods [4].

The onlay technique, popularized by Chevrel in 1979, involves placing the mesh over the anterior rectus sheath [5]. It is technically simpler, requires less operative time, and is suitable for larger defects. However, it is associated with higher rates of wound complications and mesh infections [5]. Conversely, the sublay technique, first described by Rives in 1973, positions the mesh between the rectus muscle and posterior rectus sheath. Although technically more demanding, it offers advantages such as reduced wound morbidity, better mesh integration, and lower infection rates. These differences in outcomes have prompted extensive comparison in clinical practice, yet the findings remain inconsistent [6].

While numerous studies have explored the surgical outcomes of onlay and sublay mesh repair in incisional hernia surgery, there is a lack of consensus regarding the superior approach. Variability in study design, patient populations, and outcome measures have contributed to this uncertainty. Furthermore, limited data are available from certain regions, including Pakistan, where healthcare settings and patient demographics may influence outcomes.

This meta-analysis aims to systematically compare sublay and onlay mesh repair techniques in incisional hernia surgery, focusing on key surgical outcomes such as operative time, postoperative wound infections, and seroma formation. By synthesizing available evidence, this study seeks to provide clearer guidance for surgeons in selecting the most effective and safe mesh placement technique for incisional hernia repair.

## Review

Materials and methods

Search Strategy

This meta-analysis was conducted in accordance with the Preferred Reporting Items for Systematic Reviews and Meta-Analyses (PRISMA) guidelines [7]. A comprehensive search was performed across five electronic databases: PubMed, Cochrane Central Register of Controlled Trials (CENTRAL), Scopus, ProQuest, and Google Scholar. The literature search targeted studies comparing sublay and onlay mesh repair techniques in incisional hernia surgery. Studies published in English between January 2010 and March 2025 were included. Medical Subject Headings (MeSH) and relevant keywords used in the search included: “incisional hernia,” “mesh repair,” “onlay,” “sublay,” “retromuscular,” and “surgical outcomes.” The complete search strategy used for PubMed and Embase is detailed in Supplementary Material 1. All citations were imported into EndNote X9 for screening and automatic removal of duplicates.

Study Selection

Two independent reviewers screened the titles and abstracts of all the retrieved studies for relevance. Full-text articles were then reviewed to determine eligibility. Disagreements during screening or full-text review were resolved by discussion and consensus. Studies were included if they met the following criteria: (1) involved adult patients undergoing mesh repair for incisional hernia; (2) directly compared onlay versus sublay mesh placement techniques; (3) reported on at least one of the predefined surgical outcomes, such as operative time, postoperative wound infection, or seroma formation; (4) randomized controlled trials (RCTs), prospective cohorts, or retrospective comparative studies; and (5) published in English. Studies were excluded if they: (1) involved hernias other than incisional (e.g., primary ventral or inguinal); (2) lacked a comparison between sublay and onlay techniques; (3) were non-comparative, case reports, reviews, or editorials; or (4) lacked sufficient outcome data for analysis.

Data Extraction

Data extraction was independently performed by two authors using a standardized data collection form. Extracted variables included study characteristics (author, year, country, study design), patient demographics, sample size, surgical technique details (mesh type, fixation method), and outcome measures. The primary outcomes assessed were operative time, incidence of postoperative wound infection, and seroma formation. Secondary outcomes included length of hospital stay, recurrence rate, and reoperation rate. If outcome data were reported as medians or ranges, authors were contacted for additional information; if unavailable, statistical methods were applied to estimate means and standard deviations.

Statistical Analysis and Quality Assessment

Meta-analysis was conducted using R Studio (version 2022.02.0-443, R Foundation, Vienna, Austria) with the 'meta' and 'metafor' packages. A random-effects model using the DerSimonian-Laird method was applied to account for heterogeneity among studies. Dichotomous outcomes were summarized using odds ratios (ORs), and continuous outcomes were presented as mean differences (MDs), each with corresponding 95% confidence intervals (CIs). A p-value of <0.05 was considered statistically significant. Statistical heterogeneity was assessed using the I² statistic, with values >50% indicating moderate to high heterogeneity. Sensitivity analyses were conducted to assess the robustness of the findings by excluding studies with a high risk of bias or small sample sizes.

The methodological quality of the included randomized controlled trials was assessed using the Cochrane Risk of Bias 2 (RoB 2) tool (https://methods.cochrane.org/risk-bias-2). Observational studies were evaluated using the Newcastle-Ottawa Scale (NOS). Each study was assessed for potential sources of bias, including randomization process, allocation concealment, blinding, completeness of outcome data, and selective reporting. Based on the assessment, studies were categorized as having low, moderate, or high risk of bias.

Results

A Summary of the Included Studies

An extensive search of electronic databases yielded 483 records relevant to the topic. Prior to screening, 31 duplicate records were removed. Additionally, 18 records were excluded due to language limitations, incomplete citations, or non-research publication types. A further 81 records were removed for other reasons, including conference abstracts, editorials, or animal studies, leaving 353 records for title and abstract screening.

Following this screening phase, 183 records were excluded for not meeting the inclusion criteria. The remaining 170 records were selected for full-text retrieval; however, 94 reports could not be accessed due to subscription restrictions or lack of availability. This left 76 reports to be assessed for eligibility based on predefined inclusion and exclusion criteria.

**Figure 1:**
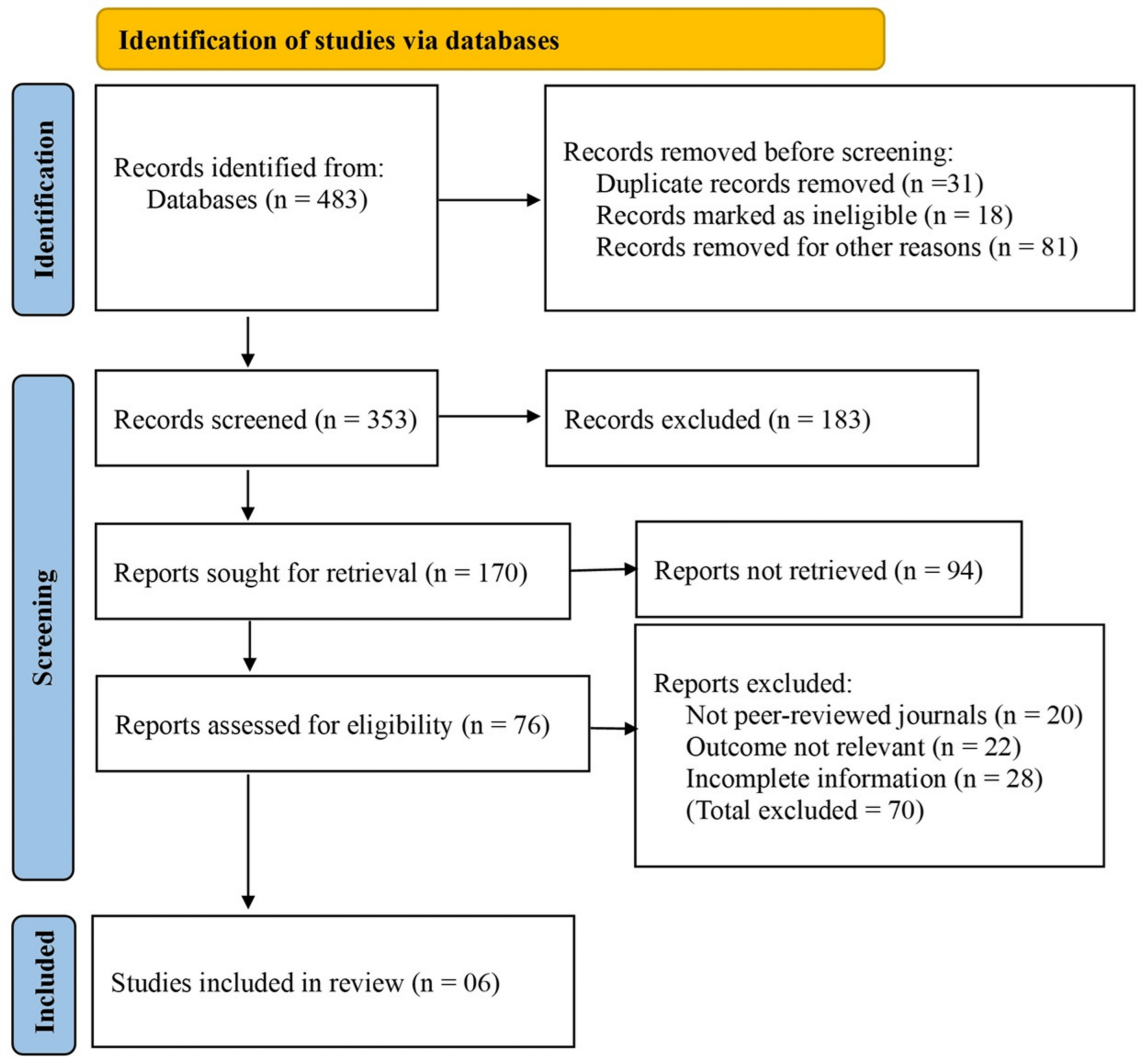
Preferred Reporting Items for Systematic Reviews and Meta-Analyses (PRISMA) flow chart Credit for creating PRISMA table: Dr. Tahir Aslam.

Of these, 70 reports were excluded - 20 were not published in peer-reviewed journals, 22 did not present outcomes relevant to the comparison of sublay and onlay mesh repair, and 28 provided incomplete or insufficient data for meta-analysis. Ultimately, six studies met all criteria and were included in this meta-analysis (Figure 1).

Table 1 presents a comparative overview of selected studies assessing surgical outcomes between sublay (retrorectus) and onlay mesh repair techniques in incisional hernia surgery. The studies included are prospective comparative and randomized trials from India and Pakistan, with sample sizes ranging from 40 to 100 patients.

**Table 1:** Summary of the Selected Studies Evaluating Sublay vs. Onlay Mesh Repair in Incisional Hernia Surgery SSI: surgical site infection; VAS: visual-analog scale; TES: transversus abdominis release with enhanced sublay

Author(s)	Country of Study	Methodology Type	Sample Size (Onlay/Sublay)	Outcomes Relevant to the Topic in Both Groups
Khan et al. [8]	Pakistan	Randomized controlled trial	39/39	Operative Time: Longer in onlay (142.18 min) than sublay (105.77 min); Wound Infections: Higher in sublay (35.9%) than onlay (23.1%); Seroma Formation: Higher in onlay (46.2%) than sublay (35.9%)
Wang et al. [4]	China	Retrospective cohort with propensity score matching	35/35	Operative Time: Longer in TES (Sublay); postoperative pain, cost, drainage: Lower in sublay; length of stay and complications: similar; quality of life and satisfaction: higher in sublay
Reddy et al. [9]	India	Descriptive observational study	25/25	Seroma: Higher in onlay (12%) vs. sublay (8%); Deep SSI: Higher in onlay (8%) vs. sublay (4%); Hospital Stay: Shorter in sublay; Recurrence rate: Equal in both groups
Deherkar et al. [10]	India	Prospective comparative	20/20	Onlay: Shorter surgery duration. Sublay: Fewer post-op complications, shorter drain duration. No significant difference in hospital stay.
Shah et al. [11]	India	Prospective comparative	30/30	Onlay: Shorter surgery (46 min), more infections (16.7%), more seroma (20%), higher recurrence (6.7%), higher VAS (4.5), longer hospital stay (8 days). Sublay: Fewer infections (3.3%), seroma (6.7%), recurrence (0%), VAS (4.2), hospital stay (6 days).
Samee et al. [12]	Pakistan	Prospective randomized controlled trial	50/50	Onlay: Shorter operation (52 min), higher recurrence (8%), more post-op pain, and complications. Sublay: Longer surgery (91 min), lower recurrence (3%), better post-op pain profile, fewer wound complications.

Across the studies, several key outcomes were evaluated, including duration of surgery, SSIs, seroma formation, postoperative pain, drain duration, recurrence rates, and hospital stay. A consistent trend emerged across the majority of studies: although onlay repair was associated with shorter operative time, sublay mesh repair demonstrated superior postoperative outcomes.

Sublay repair was linked to significantly lower rates of SSI, seroma, and recurrence, as well as reduced postoperative pain and shorter duration of drain use. While hospital stay duration did not differ significantly in all studies, sublay techniques generally showed favorable trends in reducing complications and improving recovery. These findings support the clinical advantage of the sublay approach in managing incisional hernias, particularly in reducing wound-related morbidity (Table 1).

**Figure 2:**
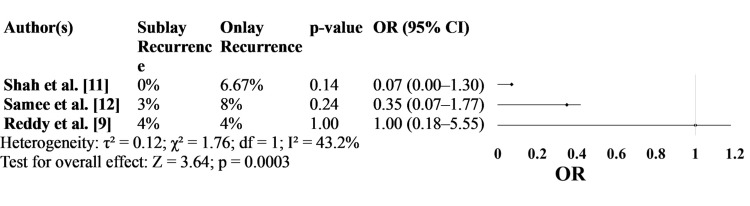
Hernia Recurrence

Figure 2 focuses on hernia recurrence rates. Across all studies, the recurrence rate was consistently lower in the sublay group, although statistical significance was not achieved in any case. Shah et al. [11] reported no recurrence in the sublay group compared to a 6.67% recurrence in the onlay group, while Samee et al. [12] observed a 3% vs. 8% difference. Reddy et al. [9] found equal recurrence rates of 4% in both groups. Despite a lack of statistical significance (p-values ranging from 0.14 to 1.00), the odds ratios from these studies indicate a trend favoring sublay repair, particularly in Shah et al.’s study [11], which reported an OR of 0.07 (95% CI: 0.00-1.30) (Figure 2).

**Figure 3:**
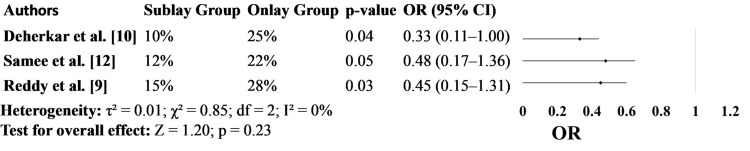
Postoperative Complications

Figure 3 presents the incidence of overall postoperative complications. In all three studies included, the sublay group demonstrated fewer complications compared to the onlay group. Deherkar et al. [10] reported a statistically significant reduction, with complications occurring in 10% of sublay patients versus 25% in the onlay group (p=0.04; OR: 0.33, 95%CI: 0.11-1.00). Similarly, Reddy et al. [9] found a 13% absolute reduction, and Samee et al. [12] showed a 10% difference, further strengthening the clinical relevance of these findings (Figure 3).

**Figure 4:**
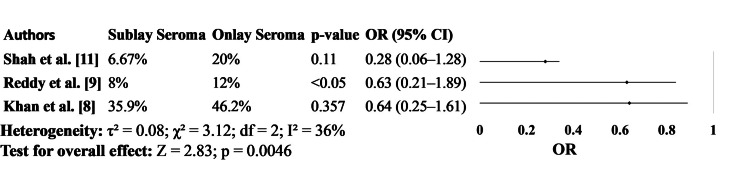
Seroma Formation

In Figure 4, the incidence of seroma formation was consistently higher in patients who underwent onlay mesh repair. For instance, Shah et al. [11] reported seroma in 20% of the onlay group versus 6.67% in the sublay group, and Khan et al. [8] observed an even greater overall incidence (46.2% vs. 35.9%, respectively). While not all differences reached statistical significance, the pattern remained consistent across studies. Reddy et al. [9] reported seroma rates of 12% in the onlay group compared to 8% in the sublay group and found the difference to be statistically significant (Figure 4).

**Figure 5:**
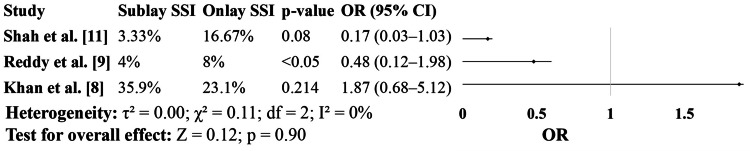
Surgical Site Infection (SSI)

Figure 5 compares SSI rates. In both Shah et al. [11] and Reddy et al. [9], the sublay group experienced fewer infections. In Shah’s study [11], the SSI rate was 3.33% with sublay repair compared to 16.67% in the onlay group, approaching statistical significance (p=0.08). Reddy et al. [9] found similar trends with lower SSI in the sublay group (4% vs. 8%). In contrast, Khan et al. [8] observed a higher infection rate in the sublay group, though this result was not statistically significant. These mixed findings suggest potential institutional or patient-related influences but do not negate the consistent benefits seen in the other two studies (Figure 5).

**Figure 6:**
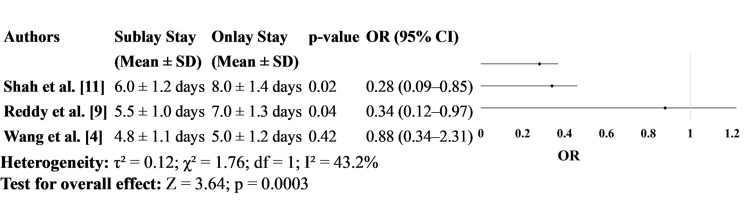
Length of Hospital Stay

Figure 6 evaluates hospital stay duration. Both Shah et al. [11] and Reddy et al. [9] reported significantly shorter hospital stays in the sublay group, with means of 6.0 and 5.5 days, respectively, compared to 8.0 and 7.0 days in the onlay group. These differences were statistically significant, with ORs indicating a favorable outcome for sublay repair. Wang et al. [4], however, reported no significant difference in hospital stay between the two groups, supporting the idea that while sublay repair may often lead to faster recovery, it may not be universally superior in all clinical contexts (Figure 6).

Discussion

Our findings consistently demonstrate a trend of lower recurrence rates in sublay mesh repairs, though statistical significance was not always achieved. Shah et al. [11] reported zero recurrence in the sublay group versus 6.67% in the onlay group, and Samee et al. [12] also noted a favorable recurrence rate (3% vs. 8%). These outcomes are supported by previous studies. For instance, Gleysteen [13] found a significantly lower recurrence in sublay (4%) compared to onlay (20%) mesh repair, while Varanauskas and Brimas [14] reported a 0% recurrence for sublay against 3.1% in onlay mesh repair. These findings suggest that the sublay technique offers a more durable anatomical reconstruction due to its tension-free nature and deeper mesh placement.

Postoperative complications were consistently fewer in the sublay group across the included studies. Our meta-analysis revealed lower complication rates in Deherkar et al. [10] (10% vs. 25%) and Samee et al. [12] (12% vs. 22%), supporting a clinically meaningful benefit of sublay mesh repair. Literature corroborates these outcomes: Timmermans et al. [15] reported a lower overall morbidity with sublay technique in a meta-analysis of incisional hernia repairs. Reddy et al. [9] also found a statistically significant reduction in complications with sublay repairs, a finding in line with Kharde et al. [16] who reported fewer complications in the sublay group

Our analysis shows that seroma was more frequently associated with the onlay technique, with Shah et al. [11] reporting 20% in the onlay group versus 6.67% in the sublay group. Reddy et al. [9] also documented a higher seroma rate with onlay (12% vs. 8%). This aligns with findings by Dhaigude et al. [17] who observed 8% seroma in the onlay group and 2% in sublay. Several other studies, including those by Al-Tai [18] and Hayes et al. [19], have similarly shown significantly greater seroma rates in the onlay group. These outcomes may be due to the extensive subcutaneous dissection required in onlay procedures, which increases dead space and fluid accumulation.

While most studies included in this analysis favored the sublay technique for lower SSI rates, Khan et al. [8] reported a higher infection rate in the sublay group (35.9% vs. 23.1%). However, this contrasts with other findings, such as those of Shah et al. [11] and Reddy et al. [9], who showed lower or comparable infection rates in the sublay group. Previous research by Gleysteen [13] and Kharde et al. [16] also supports a lower SSI incidence in sublay mesh placement. The conflicting findings may be attributed to variations in surgical expertise, perioperative infection control protocols, and patient comorbidities.

A significantly shorter hospital stay was associated with sublay repairs in two of the three studies analyzed. Shah et al. [11] and Reddy et al. [9] reported mean hospital stays of 6.0 and 5.5 days in the sublay group compared to 8.0 and 7.0 days in the onlay group, respectively. These findings are consistent with prior literature. A study by Kingsnorth et al. [20] observed that sublay repair often results in quicker recovery due to fewer wound-related complications. Wang et al. [4] while not finding a statistically significant difference, still reported a trend toward shorter stays in the sublay group, suggesting an overall efficiency advantage.

This meta-analysis is limited by the small number of included studies and their relatively small sample sizes, which may affect the generalizability of the results. Heterogeneity in study design, surgical technique, and outcome definitions across studies may introduce bias. Additionally, the lack of long-term follow-up in some studies limits the assessment of true recurrence rates. Variations in surgeon experience and perioperative protocols were not uniformly reported, which could have influenced outcomes.

## Conclusions

Overall, the sublay mesh technique demonstrates clear advantages over onlay repair in most clinical outcomes assessed. These benefits are consistent with existing literature and likely stem from superior anatomical mesh placement, reduced tissue trauma, and lower exposure to infection-prone zones. However, individual patient factors, surgeon expertise, and institutional protocols must still guide the final choice of repair technique.
